# Computational simulation of the effects of oxygen on the electronic states of hydrogenated 3C-porous SiC

**DOI:** 10.1186/1556-276X-7-471

**Published:** 2012-08-22

**Authors:** Alejandro Trejo, Marbella Calvino, Estrella Ramos, Miguel Cruz-Irisson

**Affiliations:** 1 Instituto Politécnico Nacional, ESIME-Culhuacan, Av. Santa Ana 1000, Mexico, DF 04430, Mexico; 2 Instituto de Investigaciones en Materiales, Universidad Nacional Autónoma de México, A. P. 70-360, Mexico, DF 04510, Mexico

**Keywords:** Porous silicon carbide, DFT, Oxygenation, Surface passivation, Porous nanostructures, Electronic properties, 81.05.Rm, 31.15.A-, 61.50.Lt

## Abstract

A computational study of the dependence of the electronic band structure and density of states on the chemical surface passivation of cubic porous silicon carbide (pSiC) was performed using *ab initio* density functional theory and the supercell method. The effects of the porosity and the surface chemistry composition on the energetic stability of pSiC were also investigated. The porous structures were modeled by removing atoms in the [001] direction to produce two different surface chemistries: one fully composed of silicon atoms and one composed of only carbon atoms. The changes in the electronic states of the porous structures as a function of the oxygen (O) content at the surface were studied. Specifically, the oxygen content was increased by replacing pairs of hydrogen (H) atoms on the pore surface with O atoms attached to the surface via either a double bond (X = O) or a bridge bond (X-O-X, X = Si or C). The calculations show that for the fully H-passivated surfaces, the forbidden energy band is larger for the C-rich phase than for the Si-rich phase. For the partially oxygenated Si-rich surfaces, the band gap behavior depends on the O bond type. The energy gap increases as the number of O atoms increases in the supercell if the O atoms are bridge-bonded, whereas the band gap energy does not exhibit a clear trend if O is double-bonded to the surface. In all cases, the gradual oxygenation decreases the band gap of the C-rich surface due to the presence of trap-like states.

## Background

Nanoscale engineering of silicon carbide (SiC) allows for considerable modification of its basic physicochemical properties. For example, SiC nanostructures have shown greater elasticity and strength than bulk SiC [1], and SiC nanowires have stable emission properties and an electron field emission threshold comparable to those of carbon nanotube-based materials. Various SiC nanostructures, such as nanospheres, nanowires, nanorods, nanopowders, and even nanoflowers, have been developed [2-5] with interesting technological applications. Among the multiple SiC nanostructures, porous silicon carbide (pSiC) is particularly interesting for the development of engineering technologies and applications, such as LEDs, photodetectors, and hydrocarbon gas sensors [6], because of its high strength and hardness, low expansion coefficient, chemical and thermal stability at elevated temperatures, and good thermal shock resistance and thermal conductivity [7]. Other interesting applications of pSiC can be found in the biotechnology field, where pSiC could be used as a membrane in implantable biosensors because it exhibits less protein adhesion than porous silicon [8]. Additionally, pSiC exhibits highly efficient blue-to-violet photoluminescence at room temperature [9], which makes it suitable for optoelectronic applications.

SiC is a material with multiple polytypes, and porous structures, such as 6H and 4H porous SiC, have been developed in these polytypes. The cubic SiC (3C-SiC) porous structures have not received as much attention because of the special conditions required to grow crystals of this polytype [10]; however, cubic pSiC exhibits promising properties for use in fast-response hydrogen (H) sensors [11]. However, only a few theoretical investigations of the electronic structure of 3C-pSiC have been reported. For example, in our previous work [12], we addressed the effects of quantum confinement and, to a lesser scale, the effect of the C or Si richness in the surface of H-terminated pSiC on the electronic structure. In addition, we studied the effect of oxygen (O) atoms on the surface passivation of pSiC with mixed surface configurations [13]. However, we did not perform a detailed study on the effects of O at the pSiC surface with only C or Si atoms, or a study on the difference between a bridge bond (X-O-X, X = Si or C) and an O double bond at the surface of the pores. This research could be fundamental to understanding the properties of these materials to enhance their possible applications.

Motivated by the experimental development of the synthesis and characterization of pSiC [11], we performed a study of the structural and electronic properties of pSiC using density functional theory (DFT) as described by the generalized gradient approximation. In particular, we have used a revised version of the Perdew-Burke-Ernzerhof (RPBE) exchange-correlation functional. The energetic stability, structure, and dispersion of the electronic states were investigated for full surface passivation of the dangling bonds with H and, as a first model of surface oxidation, for surfaces created by replacing H with O atoms in certain positions at the pore surface. Two different types of bonding, including double and single bonds between O and Si or C atoms, were explored.

## Methods

**Figure 1:**
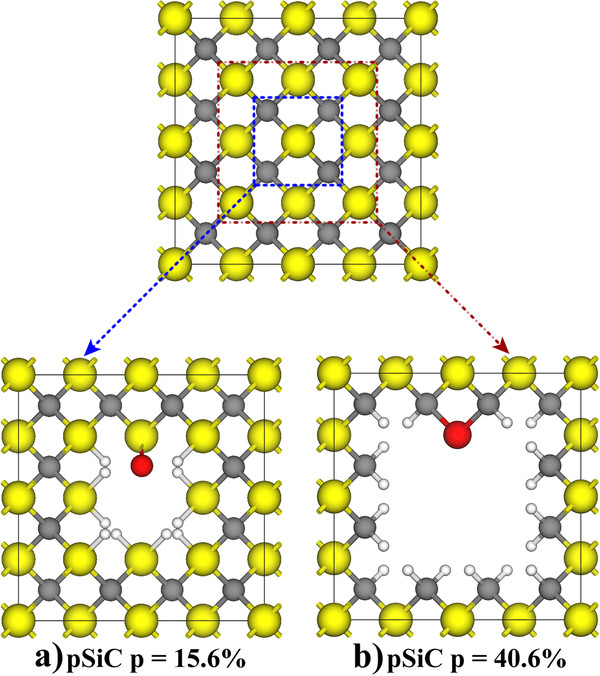
**Porous silicon carbide model.** In the upper panel, the original 32-atom supercell is shown. The squares represent the atoms removed to create the porous structures depicted. The lower panels present pSiC with (**a**) a Si-rich surface with a double-bonded O and (**b**) a C-rich surface with a bridge-bonded O. The yellow, gray, light-gray, and red spheres represent Si, C, H, and O atoms, respectively.

The porous structures were modeled using the supercell scheme described elsewhere [13], where Si and C atoms were removed in the [001] direction of an otherwise perfect 3C-SiC crystal. The porosity (p) was defined as the ratio between the removed atoms and the original number of atoms in the supercell. A 32-atom supercell was chosen to model pores of different sizes and chemical surface compositions, as shown in Figure 1. Because of the binary nature of SiC, it is possible to obtain multiple surface configurations. As a first approach, we focused on two particular cases in which the pore surfaces were exclusively composed of Si or C atoms (Si-rich and C-rich surfaces, respectively). To this end, we constructed two kinds of pores, each with the aforementioned surface configurations. One pore was modeled by removing 4C + 1Si atoms to create a Si-rich pore with 15.6% porosity (Figure 1a). The C-rich pore with the same porosity was constructed in a similar manner. In addition, to study the effects of quantum confinement in these structures, we modeled another pore by removing 9C + 4Si atoms, which created a Si-rich pore with 40.6% porosity, and a similar procedure was followed to create the analogous C-rich surface (Figure 1b). All surface dangling bonds were passivated with H atoms at first.

Because of the large surface area of pSiC, there are many potential sites for the attachment of various chemical species, such as O atoms, which were studied in this work. We analyzed the effects of the interaction of O atoms with the pore surface on the electronic structure by replacing pairs of H atoms on the passivated surface with O atoms. The changes in the electronic structure that arise due to two types of O bonding environments at the surface, single Si-O-Si or C-O-C bonds (bridge-bonded) and double Si = O or C = O bonds, were compared. The calculations of the electronic band structure and density of states (DOS) of pSiC were performed using an *ab initio* density functional theory generalized gradient approximation scheme as described by a revised version of the RPBE [14] functional and norm-conserving pseudopotentials [15] as implemented in the CASTEP code [16]. We used a cutoff energy of 750 eV and a highly converged *k*-point set according to the Monkhorst-Pack scheme [17] with grids of up to 3 × 3 × 6 *k* points. All structures were optimized to their basal state by modifying the atomic coordinates and the supercell shape through the BFGS algorithm [18]. DFT is known to underestimate the band gap energy. This problem can be overcome using other approximations, such as GW; however, in this work, we focused on the relative energetic stability and electronic property differences between the pores and, hence, did not apply any correction.

## Results and discussion

**Figure 2:**
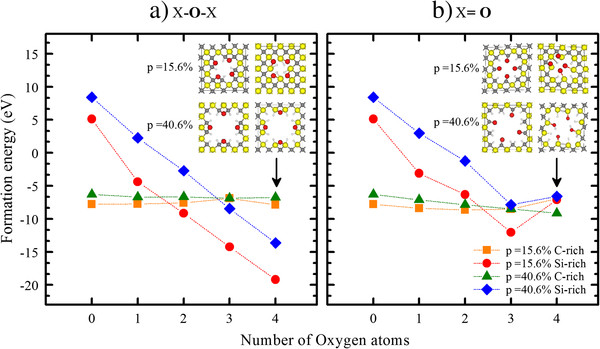
**Formation energy evolution with increasing O levels.** The formation energies for the (**a**) double-bonded and (**b**) bridge-bonded cases are shown. In the insets, the 4-oxygen structures for each surface are shown in both cases. The squares and solid circles represent the 15.6% porosity results, and the triangles and diamonds represent the 40.6% porosity results.

To study the stability of the pSiC structures with different porosities and varying amounts of oxygenation, we calculated the formation energies *E*_f_ according to the expression [19]:

(1) $$E_{\text{f}}=E_{\text{pSiC}}-\sum_{i=\text{Si,C,H,O}}n_{i}\mu_{i}$$

where *E*_pSiC_ is the ground-state energy of the passivated pSiC, *n*_i_ indicates the number of the atomic species per supercell, and *μ*_i_ is the chemical potential of the atomic species. Other quantities also influence the value of the formation energies (such as the zero-point vibrational energy and the pressure and temperature dependence of the chemical potentials); nevertheless, this approximation can give reasonable estimations of the stabilities of the structures. The chemical potentials of Si and C were calculated for their respective diamond fcc structures, whereas the O and H chemical potentials were calculated for their molecular states (O_2_ and H_2_) using the same parameters as described in the previous section. Figure 2 shows the evolution of the formation energy as a function of the number of O atoms present on the surface of the pores. The results of the formation energy calculations show that the most stable configuration corresponds to the 4-oxygen bridge-bonded Si-rich pSiC with a porosity of 15.6%, followed by the 3-oxygen bridge-bonded case of the same structure and the 4-oxygen bridge-bonded pSiC with a porosity of 40.6%. These results might be due to the tendency of the O bridge bonds to rearrange to a structure similar to that of the highly stable α-quartz SiO_2_ by modifying the initial Si-O-Si bond angle and length to those described by Lager [20]. Another result that can be observed in Figure 2a is that an increase in the number of O bridge bonds at the Si-rich surface leads to a decrease in the formation energy. In contrast, this behavior is not observed for the C-rich phase, which maintains an almost constant energy formation value irrespective of the oxygen ratio at the surface. This finding supports the hypothesis that the decrease in the formation energy and, hence, the increase in the stability of the Si-rich structures are due to the presence of Si-O-Si bonds. The high stability of the systems with Si-O-Si bonds at the pore surface supports some experimental results [21] that show the formation of a SiO_2_ phase at the pore surface of pSiC under specific experimental conditions. The negative formation energies of the C-O-C systems indicate that these systems are also energetically favorable.

For the double-bonded oxygen systems, a decrease in the formation energy with increasing amounts of oxygen at the surface that is similar to that observed in the bridge-bonded cases shown in Figure 2b is observed. However, when four oxygen atoms are present at the surface, an increase in the formation energy is observed with the calculated energies for both porosities almost converging at the same value. This behavior can be attributed to the large lattice deformations caused by the O atom interactions in the 15.6% porosity case. This structure shows that it is possible for an O_2_ molecule to be released during the formation of this pore (inset in Figure 2b). Notably, after structural relaxation, the double-bond character is lost at almost all Si-rich surfaces, and a tendency to create Si-O-Si bridge bonds is observed instead. The double-bond character is preserved in all of the C-rich surface cases along with the original symmetry, which indicates the stability of this type of bond at the pore surface. The energetically favorable formation energies of the systems with C = O bonds also support the results of some experimental studies, which suggest that C oxides form at the pore surface [21] in contrast to the SiC/SiO_2_ interface and mixed oxides that are mainly formed in the bulk [22]. A slight increase in the formation energy of the 4-oxygen double-bonded C-rich surface is observed and is explained in terms of the surface bond distortions in the proximity of the C-H bonds due to the presence of the neighboring C = O double bonds.

In addition, the replacement of H atoms with O atoms can be regarded as a defect in the surface passivation of H-terminated pSiC. To study the preferential configurations of O insertion at the passivated pSiC surface, we calculated the defect formation energy, which is defined as the energy needed to attach an O atom at the pore surface after removing the H atoms, using the following expression [23-25]:

(2) $$\Omega =E_{\text{O-pSiC}}-E_{\text{H-pSiC}}+m\mu_{\text{H}}-n\mu_{\text{O}}$$

where Ω denotes the defect formation energy, *E*_O-pSiC_ and *E*_H-pSiC_ are the total energies of the oxygenated and hydrogenated pSiC, respectively, *m* is the number of H atoms removed per supercell, *n* is the number of O atoms added per supercell, and *μ*_H_ and *μ*_O_ are the chemical potentials of O and H, respectively, as previously calculated. The results of the defect formation energy calculations for all porous cases are summarized in Table 1.

**Table 1:** Calculated results of Ω (in eV) for O defects in the H-pSiC passivated surface

**Porosity (%)**	**O atoms added per supercell (*n*)**	**H atoms removed per supercell (*m*)**	**Defect formation energy (Ω)**			
			**C-rich**		**Si-rich**	
			**C-O-C**	**C = O**	**Si-O-Si**	**Si = O**
15.6	1	2	0.048	−0.606	−9.509	−8.227
	2	4	0.199	−0.866	−14.264	−11.443
	3	6	0.887	−0.741	−19.364	−17.158
	4	8	−0.08	0.841	−24.308	−12.248
40.6	1	2	−0.4	−0.828	−6.145	−5.438
	2	4	−0.391	−1.567	−11.134	−9.658
	3	6	−0.585	−2.195	−16.866	−16.266
	4	8	−0.452	−2.853	−22.056	−15.007

The preferential configurations involve bridge-bonded O species at the Si-rich pSiC surface because these configurations have the lowest defect formation energy values. The most stable bridge-bonded substitution in the Si-rich pores is the one with four O atoms replacing eight H atoms for both porosities, as shown by their defect formation energies (−24.308 eV for 15.6% porosity and −22.056 eV for 40.6% porosity). These results indicate that as the number of bridge-bonded O atoms replacing H atoms increases, less energy is needed to stabilize the defects in the passivation, most likely because fewer interactions occur between O and H. For the Si-rich double-bonded substitution case, although all the defects in the surface passivation are energetically stable, the defect formation energies do not decrease monotonically. A sharp increase is observed in the formation energy for the 4-oxygen case, which might be due to interactions between H and O atoms at the surface of the nanopores that can lead to the creation of OH groups and a concomitant change in the surface chemistry of the pores. These changes in the surface structure mean that the formation energy might represent a third type of defect in the passivation. Notably, the deformation energy of the 40.6% porosity case is lower than that of the 15.6% case for the 4-oxygen double-bonded substitution at the Si-rich surface, in contrast to the trend observed for all other Si-rich surfaces. This particular shift might also be due to the formation of OH defects, which change the formation energy behavior, instead of the double-bonded O defects that are preserved to some degree in the 15.6% case.

In the C-rich case, the defect formation energy is almost constant for all surfaces, with the double-bonded defects being more energetically favorable than the bridge-bonded defects, in contrast to the Si-rich case. The greater energetic values of the C-rich surfaces compared to those of the Si-rich surfaces show that the inclusion of O at the C-rich surface is energetically less favorable, most likely in part due to a stronger C-H bond, which requires more energy to be broken than the Si-H bond. However, the double-bonded impurities seem to be stabilized better by the 40.6% porosity than by the 15.6% porosity, which suggests that greater stabilization of these impurities in the surface passivation of C-rich pSiC is possible at larger porosities.

**Figure 3:**
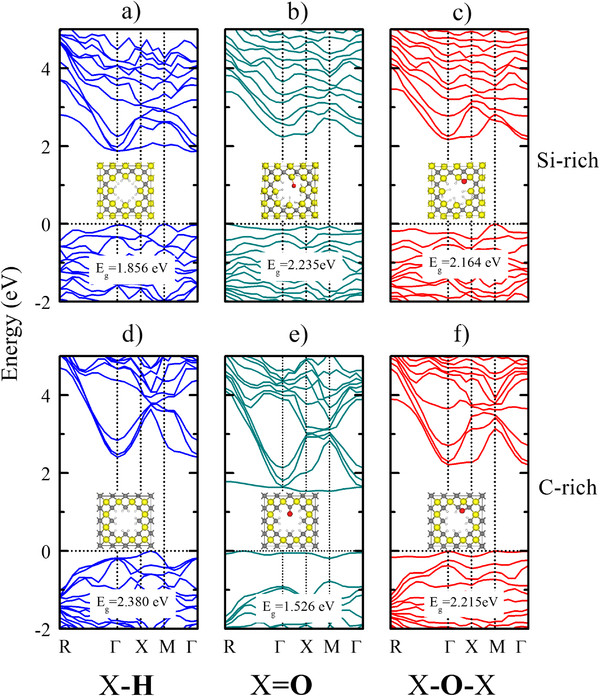
**Electronic band structure of pSiC with different passivation schemes.** The electronic band structures of the Si-rich pSiC surfaces with (**a**) H passivation and 1-oxygen (**b**) double-bonded and (**c**) bridge-bonded at the surface are shown. The electronic band structures of the C-rich surfaces with (**d**) H passivation and 1-oxygen (**e**) double-bonded and (**f**) bridge-bonded at the surface are also displayed. In the inset in each panel, we show the optimized pSiC structures for which the band structures were calculated.

Figure 3 shows the electronic band structure of H-passivated pSiC with a porosity of 15.6% and a comparison of it to those of the 1-oxygen bridge-bonded and double-bonded surfaces for both the Si-rich and C-rich cases. The Si surface exhibits a smaller band gap than the C surface, which can be explained in terms of the lower bond energy of Si-H compared to that of C-H for their corresponding molecules [26]. The effect of replacing a pair of H atoms with one O atom in the Si surface case can be observed in Figure 3b,c. For the double bond and the bridge bond, a significant band gap broadening is observed. As shown in the inset in Figure 3b, the double-bond configuration relaxes to a geometry similar to that of the bridge-bonded configuration (inset in Figure 3c), which is consistent with the greater stability of the Si-O-Si bond. Hence, the band gap broadening in both cases might have the same explanation. One hypothesis is that the greater polarity of the Si-O bond compared to that of the Si-H bond results in a decrease in the energy of the electronic levels, especially for the valence band. However, the same comparison is made for the 100% C surface case, and different results are obtained, as shown in Figure 3e,f. At the 1-oxygen double-bond surface, two bands appear near the prohibited band gap and are attributed to the extra *p* states that arise due to the planar nature of the C = O bond. This geometry involves sp^2^-hybridized orbitals, and the π bond is constructed from *p* orbitals on both O and C, which are the main contributors to the orbitals at the edges of the band gap. The bridge-bonded O surface (Figure 3f) has a larger band gap than the double-bonded O surface because of the tetrahedral geometry at the surface near the C-O-C bond, which involves an sp^3^-like hybridization.

**Figure 4:**
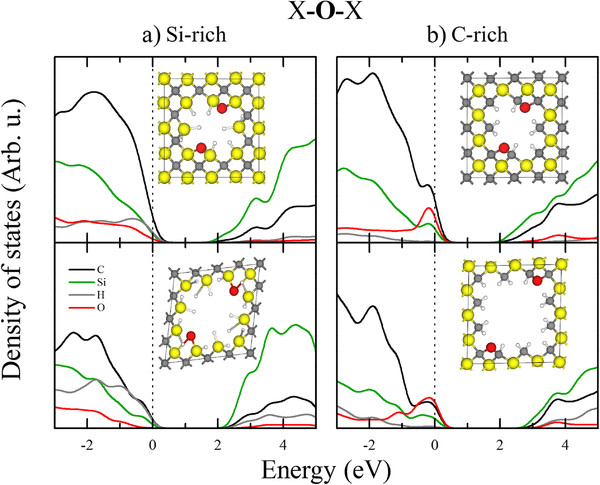
**Local density of states of pSiC.** LDOS of the 2-oxygen bridge-bonded porous SiC with (**a**) Si- and (**b**) C-rich surfaces with 15.6% (top panels) and 40.6% (lower panels) porosities are shown. The black, green, gray, and red lines represent the LDOS of C, Si, H, and O atoms, respectively. In the inset, the relaxed atomic structure model used to calculate each DOS is shown.

To investigate the quantum confinement effects and the contribution of the atoms to the band gap, we performed local density of states (LDOS) calculations in which the DOS is projected over atoms of interest in all structures. The results for the bridge-bonded case at both porosities (15.6% and 40.6%) are shown in Figure 4a,b. For the Si-rich and C-rich surfaces, the band gap increases as the porosity increases, despite the presence of the extra states introduced by the O atom. This result is consistent with a quantum confinement scheme. Notably, the higher porosity has a higher formation energy, which means that more energy is necessary to create a highly porous structure with greater confinement. For the Si-rich surfaces (Figure 4a), a large contribution from the C atoms is observed near the valence band maximum (VBM) for both porosities, whereas a major contribution from the Si atoms can be observed near the conduction band minimum (CBM). These results suggest the presence of a donor-acceptor system in which the C atoms are the donors and the Si atoms are the acceptors. C acts as the donor because its electronegativity is greater than that of Si. For the C-rich surface (Figure 4b), the behavior is similar; however, the principal contribution to the states near the VBM is from the O atoms in conjunction with the C atoms, which leads us to believe that these trap-like states are caused by the C-O-C bonds at the surface of the pores [27].

**Figure 5:**
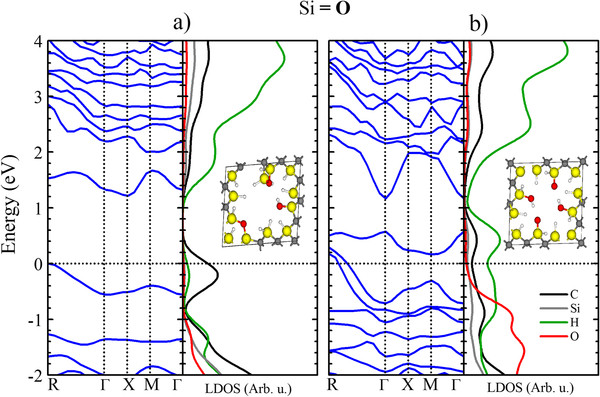
**Electronic band structure and density of states of the oxygenated Si-rich pSiC surface.** We show the electronic band structures (left panel) and densities of states (right panel) of the (**a**) 3-oxygen and (**b**) 4-oxygen surfaces; the insets illustrate the corresponding optimized atomic structure models. For the LDOS, the black, green, gray, and red lines represent the contribution of C, Si, H, and O atoms, respectively.

Finally, to study the effect of increasing the number of O atoms on the electronic states of these structures, we compared the electronic band structure and LDOS of the 3-oxygen and 4-oxygen double-bonded Si-rich pSiC surfaces, as shown in Figure 5a,b. For the 3-oxygen case, a noticeable band gap is observed, whereas the 4-oxygen surface exhibits a metallic behavior. This severe discrepancy might be caused by the formation of a Si-O-Si bridge in the 3-oxygen case, which increases the polarity of this particular bond and thus decreases the valence band levels, whereas the 4-oxygen surface has a distorted geometry, and interactions between H and O atoms at this surface (inset in Figure 5b) create dangling bond-like states in the porous structures. In all Si-rich surface pores with Si-O-Si bridging bonds, the band gap increases as the number of O atoms per supercell increases, which is consistent with the greater polarity of the Si-O-Si bond and the tendency to rearrange to the configuration of α-quartz SiO_2_. An irregular behavior in the band gap for the double-bonded cases is observed: sometimes the gap increases relative to the hydrogenated case, and sometimes, it decreases. This behavior can be explained by the rearrangement of some of the O atoms to a configuration similar to the bridge-bonded cases after the geometry optimization, which suggests the creation of bridge bonds instead of the double bonds. If the bridging bonds exist, the band gap increases; otherwise, the band gap decreases, most likely due to the distortion of the lattice caused by interactions between O atoms and neighboring H atoms that create OH radicals instead of double bonds. The OH termination has also been observed at the surface of pSiC [28]. For the C-phase surfaces, the band gap decreases as the number of O atoms per supercell increases irrespective of the type of bond. This trend is most likely due to the extra *p* states introduced by the oxygen near the conduction and valence bands from the planar (C = O) or tetrahedral (C-O-C) bonding.

**Figure 6:**
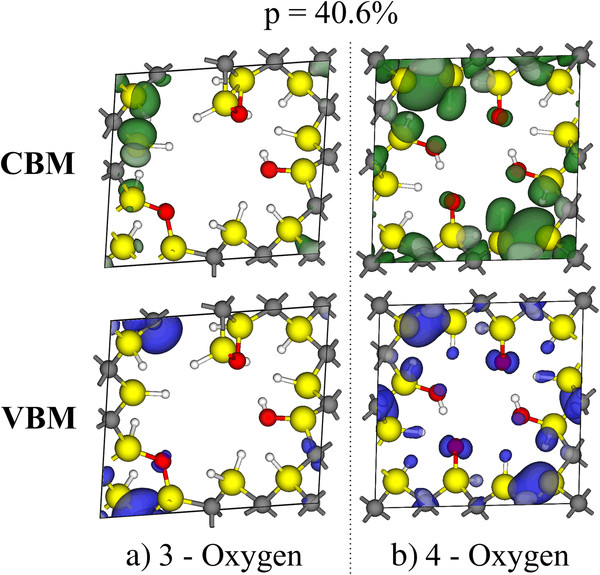
**Isosurfaces of the band gap edges of the Si-rich pSiC surface.** The VBM and CBM orbitals of the (**a**) 3-oxygen and (**b**) 4-oxygen Si-rich pSiC surfaces with 40.6% porosity are depicted. The violet (lower panels) and green (higher panel) isosurfaces represent the VBM and CBM orbitals, respectively, with an isovalue of 0.04 Å^−3^ for the 3-oxygen surface and 0.02 Å^−3^ for the 4-oxygen surface.

Figure 6a,b shows the VBM (or highest occupied molecular orbital (HOMO)) and CBM (or lowest unoccupied molecular orbital (LUMO)) orbital isosurfaces of the 3-oxygen and 4-oxygen surfaces that are illustrated in Figure 5. For the 3-oxygen surface (Figure 6a), the HOMO orbital is principally located on a C atom near a large surface deformation near the Si = O bond (Figure 6a, lower panel). The bond between this C atom and a neighboring Si atom is broken; hence, this state is caused by the dangling bond that originates from this broken bond. The LUMO is located primarily around Si atoms with dangling bonds that are formed when the attached H atoms move to replace H atoms on the Si atom that creates the pore deformation. These H atoms are likely replacing the H atoms that interact with the O atoms to form OH groups. Both observations are consistent with the information obtained from the LDOS analysis (see Figure 5). For the 4-oxygen surface (Figure 6b), an increased symmetry is observed. Both the HOMO and LUMO orbitals are located near the Si atoms to which the O atoms are attached. Based on the interactions, a rearrangement of the surface H interactions occurs, which supports the previously speculated formation of -OH groups instead of O double bonds.

## Conclusions

In summary, we have studied the energetic stability, electronic band structures, and densities of states of pSiC with different chemical surface compositions within the framework of density functional theory. The results from the energetic stability analysis show an increased energetic stability in the H-passivated Si-rich surface when some of the H atoms are replaced by O atoms, most likely due to the formation of highly stable Si-O-Si bonds. These bridging bonds form irrespective of the initial configuration of the Si-O bond. At the C-rich surface, the structures seem to lose some energetic stability; however, all porous structures are still energetically favorable. Another remarkable result is that the highly deformed structures with Si = O bonds have formation energies similar to those with C = O bonds. However, the electronic structure calculations show that the C-rich surface has a greater band gap than does the Si-rich surface when the surfaces are fully passivated by H, most likely due to the greater strength of the C-H bond compared to that of the Si-H bond. As the oxygenation of the surface increases, the bridge-bonded Si-O-Si configuration creates a broadening in the band gap energy. In contrast, no clear trend for the Si = O-bonded structures is discernible because the O atom tends to create Si-O-Si bonds in some cases and -OH groups, which reduce the band gap energy, in other cases. For all of the C-rich surfaces, the presence of the O atom reduces the band gap energy by creating trap-like states near the valence band maximum and the conduction band minimum. These states are most likely due to the extra *p* orbitals introduced by the O atoms. In comparison with our previous results [13], we see a similar behavior in the electronic band gap in the mixed Si and C surface scheme as in the bridge-bonded case. However, some key differences exist, such as the irregular changes in the band gap caused by a change in the chemical environment during the geometry optimization. The double bond is also shown to affect the band gap energy and surface constitution of the nanopores, which was not addressed previously. This result shows that there is a possibility of band gap engineering through the surface manipulation of pSiC for applications in sensors and optoelectronics.

## Competing interests

The authors declare that they have no competing interests.

## Authors’ contributions

MC performed the computer simulations and data acquisition and helped in drafting the manuscript. AT performed the formation energy calculations and the design of the study and helped in the analysis and interpretation of data and drafting of the manuscript. ER contributed to the analysis and interpretation of the structural changes and formation energy results and suggested possible explanations for the electronic structure behavior. MCI is the principal researcher who supervised this work, helped in the analysis and interpretation of data and, together with MC, AT, and ER, worked on the drafting and revisions of the manuscript. All authors read and approved the final manuscript.

## Authors’ information

AT and MC are PhD students. ER has a PhD degree in materials science and engineering and is a research associate at the Universidad Nacional Autónoma de México (UNAM). MCI has a PhD degree in materials science and is a professor at the Instituto Politécnico Nacional (IPN).

## References

[B1] Wong EW Sheehan PE Lieber CM Nanobeam mechanics: elasticity, strength, and toughness of nanorods and nanotubes Science 1997 277 1971 1975 10.1126/science.277.5334.1971

[B2] Zakharko Y Botsoa J Alekseev S Lysenko V Bluet JM Marty O Skryshevsky VA Guillot G Influence of the interfacial chemical environment on the luminescence of 3C-SiC nanoparticles J Appl Phys 2010 107 013503 10.1063/1.3273498

[B3] Fan JY Wu XL Chu PK Low-dimensional SiC nanostructures: fabrication, luminescence, and electrical properties Prog Mater Sci 2006 51 983 1031 10.1016/j.pmatsci.2006.02.001

[B4] Luo X Ma W Zhou Y Liu D Yang B Dai Y Synthesis and photoluminescence property of silicon carbide nanowires via carbothermic reduction of silica Nanoscale Res Lett 2009 5 252 256 20651911 10.1007/s11671-009-9474-8 PMC2893599

[B5] Zhang H Ding W He K Li M Synthesis and characterization of crystalline silicon carbide nanoribbons Nanoscale Res Lett 2010 5 1264 1271 10.1007/s11671-010-9635-9 20676202 PMC2897037

[B6] Kim K-S Chung G-S Characterization of porous cubic silicon carbide deposited with Pd and Pt nanoparticles as a hydrogen sensor Sens Actuators, B 2011 157 482 487 10.1016/j.snb.2011.05.004

[B7] Keffous A Bourenane K Kechouane M Gabouze N Kerdja T Guerbous L Lafane S Effect of anodization time on photoluminescence of porous thin SiC layer grown onto silicon J Lumin 2007 126 561 565 10.1016/j.jlumin.2006.10.024

[B8] Yakimova RRM Petoral J Yazdi GR Vahlberg C Spetz AL Uvdal K Surface functionalization and biomedical applications based on SiC J Phys D: Appl Phys 2007 40 6435 10.1088/0022-3727/40/20/S20

[B9] Nishimura T Miyoshi K Teramae F Iwaya M Kamiyama S Amano H Akasaki I High efficiency violet to blue light emission in porous SiC produced by anodic method Physica Status Solidi (c) 2010 7 2459 2462 10.1002/pssc.200983908

[B10] Liu L Yiu YM Sham TK Zhang L Zhang Y Electronic structures and optical properties of 6 H- and 3C-SiC microstructures and nanostructures from X-ray absorption fine structures, X-ray excited optical luminescence, and theoretical studies J Phys Chem C 2010 114 6966 6975 10.1021/jp100277s

[B11] Kim K-S Chung G-S Fast response hydrogen sensors based on palladium and platinum/porous 3C-SiC Schottky diodes Sens Actuators, B 2011 160 1232 1236 10.1016/j.snb.2011.09.054

[B12] Trejo A Calvino M Cruz-Irisson M Chemical surface passivation of 3C-SiC nanocrystals: a first-principle study Int J Quantum Chem 2010 110 2455 2461

[B13] Calvino M Trejo A Cuevas JL Carvajal E Duchén GI Cruz-Irisson M A density functional theory study of the chemical surface modification of β-SiC nanopores Mater Sci Eng, B 2012 10.1016/j.mseb.2012.02.009

[B14] Hammer B Hansen LB Nørskov JK Improved adsorption energetics within density-functional theory using revised Perdew-Burke-Ernzerhof functionals Phys Rev B 1999 59 7413 7421 10.1103/PhysRevB.59.7413

[B15] Hamann DR Schlüter M Chiang C Norm-conserving pseudopotentials Phys Rev Lett 1979 43 1494 1497 10.1103/PhysRevLett.43.1494

[B16] Clark SJ Segall MD Pickard CJ Hasnip PJ Probert MIJ Refson K Payne MC First principles methods using CASTEP Z Kristallogr 2005 220 567 570 10.1524/zkri.220.5.567.65075

[B17] Monkhorst HJ Pack JD Special points for Brillouin-zone integrations Phys Rev B 1976 13 5188 5192 10.1103/PhysRevB.13.5188

[B18] Pfrommer BG Côté M Louie SG Cohen ML Relaxation of crystals with the quasi-Newton method J Comput Phys 1997 131 233 240 10.1006/jcph.1996.5612

[B19] Aradi B Ramos LE Deák P Köhler T Bechstedt F Zhang RQ Frauenheim T Theoretical study of the chemical gap tuning in silicon nanowires Phys Rev B 2007 76 035305

[B20] Lager GA Crystal structure and thermal expansion of α-quartz SiO2 at low temperatures J Appl Phys 1982 53 6751 10.1063/1.330062

[B21] Lee K-H Lee S-K Jeon K-S Photoluminescent properties of silicon carbide and porous silicon carbide after annealing Appl Surf Sci 2009 255 4414 4420 10.1016/j.apsusc.2008.11.047

[B22] Soukiassian P Amy F Silicon carbide surface oxidation and SiO2/SiC interface formation investigated by soft X-ray synchrotron radiation J Electron Spectrosc Relat Phenom 2005 144-147 783 788

[B23] Rurali R Cartoixà X Theory of defects in one-dimensional systems: application to Al-catalized Si nanowires Nano Lett 2009 9 975 979 10.1021/nl802847p 19206213

[B24] Zhang SB Northrup JE Chemical potential dependence of defect formation energies in GaAs: application to Ga self diffusion Phys Rev Lett 1991 67 2339 2342 10.1103/PhysRevLett.67.2339 10044401

[B25] Peelaers H Partoens B Peeters FM Properties of B and P doped Ge nanowires Appl Phys Lett 2007 90 263103 10.1063/1.2752107

[B26] Cantin JL von Bardeleben HJ Ke Y Devaty RP Choyke WJ Hydrogen passivation of carbon Pb like centers at the 3C- and 4 H-SiC/SiO[sub 2] interfaces in oxidized porous SiC Appl Phys Lett 2006 88 092108 10.1063/1.2179128

[B27] Huang W-Q Jin F Wang H-X Xu L Wu K-Y Liu S-R Qin C-J Stimulated emission from trap electronic states in oxide of nanocrystal Si Appl Phys Lett 2008 92 221910 221913 10.1063/1.2937835

[B28] Fan JY Li HX Cui WN Microstructure and infrared spectral properties of porous polycrystalline and nanocrystalline cubic silicon carbide Appl Phys Lett 2009 95 021906 021903 10.1063/1.3180706

